# Postnatal development in the cold render bird mitochondria more susceptible to heat stress

**DOI:** 10.1098/rspb.2025.1027

**Published:** 2025-06-18

**Authors:** Maria Correia, Elisa Thoral, Elin Persson, Imen Chamkha, Eskil Elmér, Andreas Nord

**Affiliations:** ^1^ University of Jyväskylä, Jyväskylä, Keski-Suomi, Finland; ^2^ La Rochelle Université, La Rochelle, Nouvelle-Aquitaine, France; ^3^ Department of Biology, Lund University, Lund, Sweden; ^4^ Department of Clinical Sciences, Mitochondrial Medicine, Lund University, Lund, Sweden; ^5^ Swedish Centre for Impacts of Climate Extremes (climes), Lund University, Lund, Sweden

**Keywords:** bird, body temperature, extreme weather, heat stress, heatwave, mitochondria

## Abstract

Research on birds suggests that extreme weather events during development may have long-lasting consequences on form and function. The underlying cellular mechanisms mediating such phenotypic effects are poorly studied. We raised Japanese quail in warm (30°C) or cold (10°C) temperatures from hatching until adulthood and then measured mitochondrial metabolism in intact blood cells at representative normothermic body temperature (41°C) and a hyperthermic temperature (45°C), that quail commonly attain when heat stressed. To investigate whether any postnatal developmental effects were reversible, half of the cold- and warm-acclimated birds were assigned to a common garden (20°C) three weeks before the measurements. Across groups, hyperthermia was associated with increased proton leak but decreased phosphorylating respiration (where ATP is produced) and maximal working capacity of the mitochondria. Cold-reared birds were more strongly affected by heat stress: the increase in proton leak was 1.6-fold higher compared with warm-acclimated birds. This did not reflect developmental programming, as the difference did not remain in the common-garden birds. Our study describes the cellular consequences of overheating and suggests that cold acclimation during postnatal development is traded off against heat tolerance at the level of cellular metabolism. These findings have potential implications for understanding avian responses to climate change.

## 1. Introduction

Extreme weather events, such as heatwaves and cold spells, are predicted to increase in intensity and frequency under climate change [1] with anticipated negative effects on the fitness and survival of wildlife [2,3]. If such thermal variations are timed at sensitive windows of development (cf. [4]), evidence from mammals and birds point to potentially irreversible epigenetic modulation of morphology and physiology (e.g. [5]; reviewed by [6,7]). For example, in fowl, increased temperature during pre- or post-natal development increases peripheral circulation [8], improves evaporative cooling capacity [9], and may reduce mortality during acute heat challenges [10]. Similarly, pre- or postnatal cold stress improves thermogenic performance, attenuates the stress response and improves survival, during later-life cold challenges [11,12]. While the endocrinology of thermoregulation is reasonably well understood, also in a developmental programming perspective (reviewed by [13]), many aspects of the cellular responses linking temperature conditions in early life to subsequent performance during a thermal challenge remain understudied.

Thermoregulation in birds is achieved through a coordinate suite of behavioural and physiological processes that require energy in the form of adenosine triphosphate (ATP), which is produced almost exclusively via oxidative phosphorylation (OXPHOS) in the mitochondria [14]. OXPHOS is not, however, entirely efficient as some protons leak through the inner mitochondrial membrane (LEAK respiration) via both passive and regulated processes [15], dissipating the proton motive force as heat at the expense of reduced ATP production. Yet LEAK is a crucial aspect of mitochondrial function, forming the basis for non-shivering thermogenesis in mammalian brown adipose tissue [16] and regulating the balance between ATP production and free radical formation [17]. It follows that mitochondrial respiration is tailored to the heat or energy requirements of individuals, increasing in the cold (e.g. [18,19]) and decreasing in the warmth [20]. Additionally, research on mammals show that mitochondria are amenable to epigenetic developmental programming by environmental perturbations [21]. This suggests that alterations of the mitochondrial phenotype may be a potential avenue linking variation into developmental temperature to improved thermoregulatory control at the organismal level.

In accordance with the above, studies show that elevated embryonic temperature, or the perception of heat stress through parental signalling, causes increased baseline respiration and OXPHOS in intact blood cells in juvenile birds [22,23]. Moreover, a perinatal heat wave increased mitochondrial respiration in adolescent zebra finches (*Taeniopygia guttata*) through higher LEAK [24]. It is unclear whether this developmental programming also impacts the thermal sensitivity of mitochondria, that is, how the mitochondria subsequently *respond* to a temperature challenge. In zebra finches, acoustic heat conditioning during embryonic development did not alter the mitochondrial response to a postnatal heat challenge [22], but another study on the same species found that constant perinatal heat exposure was associated with a reduction in LEAK upon heat acclimation in adults more than 2 years after initial exposure [20]. To the best of our knowledge, however, no study on birds has addressed how the mitochondria themselves respond to a heat challenge *ex vivo* (but see [25] for the *ex vivo* response of blood cell mitochondria to cold). This data paucity is constraining the research field, not the least since most bird species respond to an acute heat challenge by (voluntary, or not) hyperthermia [26]. Therefore, studies of the thermal sensitivity of mitochondrial metabolism in a developmental temperature context could give novel insight into how animals counter extreme weather events, from cells to organisms.

To determine whether the thermal sensitivity of mitochondrial function is programmed by postnatal developmental temperature, we reared Japanese quail in simulated heatwave-like (30°C) or cold-snap-like (10°C) conditions from hatching to reproductive maturity, after which half of the individuals from each acclimation group were transferred to a common garden at intermediate temperature (20°C). Three weeks later, we measured mitochondrial respiration in intact blood cells at both a normal body temperature (41°C) and a representative hyperthermic temperature that quail incur during heat stress (45°C) (cf. [9]). We expected a higher mitochondrial respiration but lower ATP-producing efficiency, during physiological hyperthermia *ex vivo* [27,28] due to a proportionally greater increase in LEAK compared with OXPHOS. If postnatal developmental temperature impacts thermal sensitivity, we expected these changes to be attenuated in heatwave-reared birds but exaggerated in cold-reared individuals (cf. [29]). Finally, if any effect of postnatal temperature on thermal sensitivity reflected developmental programming, we expected phenotypic change to remain even when the thermal stimulus had been removed (i.e. in the common garden birds).

## 2. Material and methods

### (a) Species and housing

**Figure 1.**
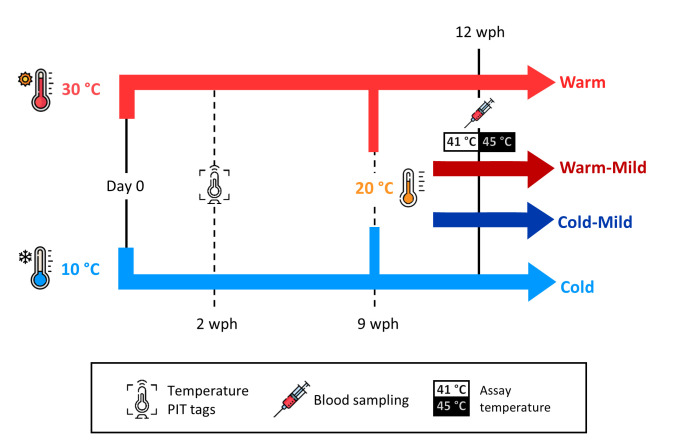
Japanese quail (*Coturnix japonica*) were reared in Warm (red, 30°C) or Cold temperature (blue, 10°C) from hatching until 9 weeks post-hatching (wph), after which half of each group was allocated to a common garden (20°C; Cold-mild group, dark blue; Warm-mild group, dark red). Birds were blood sampled at 12 wph to assess the thermal sensitivity of mitochondrial respiration, to investigate if developmental temperature programmes mitochondrial function.

The study was performed using Japanese quail (*Coturnix japonica*), a precocial bird that is reproductively active from 6 to 8 weeks of age [30]. Eggs were obtained from a commercial breeder (Sigvard Månsgård, Åstorp, Sweden) and were incubated at 37.5°C and 50% relative humidity (OvaEasy 190 Advance Series II, Brinsea, Weston, UK). A total of 55 of 85 eggs hatched (65%) and 49 chicks survived past 1 week post-hatching (henceforth, wph). On the hatching day (day 0), chicks were ringed with individual combinations of colour rings and were assigned to one of the two housing temperature treatments (figure 1): Warm group (30°C; 30.02°C ± 0.82°C; mean ± standard deviation, s.d.) or Cold group (10°C; 9.70°C ± 0.31°C). Birds were housed in groups ≤15 individuals, in open pens (310 cm × 120 cm × 60 cm) under a 14 h:10 h light:dark cycle, with food and water provided ad libitum. Until 3 wph, the birds were fed with Turkey Starter (25.5% protein; Lantmännen AB, Stockholm, Sweden), and from then onwards with Turkey Grower (22.5% protein; Lantmännen AB). Until 2 wph, hatchlings had access to a heat lamp (35°C–39°C) which was programmed to allow 6 cooldown periods during daytime (10 min every 2 h until 1 wph; 30 min every 2 h until 2 wph). The heat lamp was placed so that the quail consistently had to experience experimental room temperatures to access feed, water and cage enrichments. Birds were weighed (±0.1 g) and wing length was measured (±0.5 mm) weekly from 1 to 12 wph. At 2 wph, a temperature-sensitive passive integrated transponder tag (LifeChip BioTherm, Destron Fearing, South St Paul, MN, USA), 2.1 mm × 12 mm in size (<0.5% of body weight), was implanted into the intraperitoneal cavity under local anaesthesia (topical application of 25 mg g^−1^ lidocaine and 25 mg g^−1^ prilocaine) to measure body temperature for another study, following the procedures described by Persson *et al*. [9]. At 9 wph, half of each thermal acclimation group were randomly allocated to a common garden at intermediate temperature (20.01°C ± 0.35°C). These individuals are referred as Warm-mild and Cold-mild birds (figure 1). Final sample sizes are reported in electronic supplementary material, table S1.

### (b) Mitochondrial measurements

Blood samples (200–300 μl; i.e. 1–1.5% of the estimated total blood volume) for measurement of mitochondrial thermal sensitivity were collected from the brachial vein using a 1 ml heparinized syringe with a 29G needle at 12 wph (i.e. after 3 weeks in common-garden conditions in the Warm-mild and Cold-mild groups; see figure 1). Samples were stored cold (5°C–7°C) in 2 ml K_2_-EDTA (ethylenediaminetetraacetic acid) tubes (BD Vacutainer, Becton Dickinson AB, Plymouth, UK) until analysed 0.5−2 h later. A subset of this sample (50 + 50 μl; see below) was subsequently used to measure mitochondrial respiration and the remainder included in other assays. Previous research shows that respiration of blood cell mitochondria in birds correlates to mitochondrial respiration in tissues with more defined roles in metabolism, such as skeletal muscle [31] and the brain [23]. In some bird studies, blood respiration even correlates with organismal metabolic rate [32,33] (but see [34,35]). Additionally, human studies suggest that blood cell mitochondria respond predictably to deviations from the normal physiological status [36].

To investigate if ambient temperature during postnatal development influenced the thermal sensitivity of mitochondria, we measured mitochondrial respiration in whole blood using an Oxygraph O2k high-resolution respirometer (Oroboros Instruments, Innsbruck, Austria) following Nord *et al*. [37]. One blood sample was measured simultaneously at two assay temperatures: at 41°C, which is a representative daytime body temperature of Japanese quail [9,38], and at 45°C, which Japanese quail reach even during moderate heat stress [9]. Hence, haematological parameters and the amount of mitochondrial tissue were identical at both assay temperatures.

We used two respirometers. which were calibrated at assay temperatures (one at 41°C and one at 45°C) and at air saturation oxygen level daily. Assay temperature alternated between the instruments every day. A total of four different respirometers were used in the experiment. Mitochondrial respiration was measured in MiR05 medium (0.5 mM EGTA, 3 mM MgCl_2_, 60 mM K-lactobionate, 20 mM taurine, 10 mM KH_2_PO_4_, 20 mM HEPES, 110 mM sucrose, 1 g l^−1^ free fatty acid bovine serum albumin and pH = 7.1 [39]). Fifty microlitres of whole blood were added to 1.950 µl of MiR05 in the respirometer chamber. After closing the chamber, the baseline rate of O_2_ consumption during respiration on endogenous substrates (i.e. ‘ROUTINE’) was obtained for 10 min. Then, ATP synthase was inhibited using oligomycin (final concentration: 1 µg ml^−1^), to measure proton-leak-linked respiration (i.e. ‘LEAK’). Phosphorylating respiration, where ATP is produced (i.e. ‘OXPHOS’) was defined as the difference between ROUTINE and LEAK [39]. This was followed by the determination of the maximal uncoupled rate of respiration of the electron transport system (i.e. ‘ETS’), achieved by titration of the protonophore uncoupler carbonyl cyanide-p-trifluoromethoxy phenylhydrazone until maximum (0.5–1 µM). Last, complex I and complex III were inhibited to determine respiration of non-mitochondrial origin (i.e. ROX respiration), using rotenone (2 µM) and antimycin (1 µg ml^−1^), respectively. Since there was no further reduction in respiration when antimycin A was added on top of rotenone, ROX was defined as whichever rate was the lowest, and this value was subtracted from all other respiration rates before analyses (electronic supplementary material, table S2).

Three flux control efficiencies (FCEs) were calculated to provide information on mitochondrial function that is independent of any between-individual differences in mitochondrial content [39] (electronic supplementary material, table S2): R–L control efficiency (endogenous respiration coupled to ATP production; 1 – (LEAK/ROUTINE)), E–L coupling efficiency (coupling of the electron transport system in an excited cellular state; 1 – (LEAK/ETS)) and E–R control efficiency (indicative of aerobic scope of the electron transport system; 1 – (ROUTINE/ETS)).

To inform explanations for between-individual variation in mitochondrial respiration, we counted the total number of cells present in the sample using a TC-20 cell counter (Bio-Rad, Solna, Sweden) following Thoral *et al*. [34,35]. Additionally, we measured citrate synthase activity (a potentially useful marker of mitochondrial volume in human skeletal muscle [40]); at 41°C in a subset of samples, following Nord *et al*. [18]. Neither cell count nor citrate synthase activity were significantly related to any aspect of mitochondrial respiration in our samples (electronic supplementary material, figures S1 and S2), and so these variables were not considered further.

### (c) Statistical analyses

Statistical analyses were performed using R 4.4.1 for Windows [41].

To evaluate the response of mitochondrial respiration to a simulated increase in a heat-stressed body temperature, and whether this varies across thermal treatment groups, we analysed the effect of assay temperature (41°C and 45°C) between all treatments (Cold, Warm, Cold-mild and Warm-mild) on all respiration rates and FCEs, using linear mixed models (LMM; lmer function in the *lme4* package; [42]). Assay temperature, treatment and sex were used as fixed effects. The interaction ‘assay temperature × treatment’ was included in all models, constituting the critical test of treatment differences in the mitochondrial response to physiological hyperthermia. Bird identity was included as a random effect to account for repeated measurements on the same individual. Body mass was originally included in the models (mean-centred by sex and treatment), but since it never explained any significant variation in mitochondrial respiration (all *p* > 0.1) it was not considered further.

The effects of postnatal developmental temperature and common garden conditions on morphological features at 12 wph (body mass, wing length) were assessed using linear models (LM; lm function in the *stats* package), using treatment and sex as factors.

Estimates and standard errors for fixed effects were calculated using the emmeans() function, and slope estimates (in case of a significant interaction between assay temperature and treatment) were calculated using the emtrends() function, both from the *emmeans* package [43]. When the interaction was significant, post hoc tests were performed using the following contrasts: Cold versus Warm, Cold versus Cold-mild, Warm versus Warm-mild and Cold-mild versus Warm-mild. The significance of random effect was analysed using ranova()in the *lmerTest* package [44]. All estimates were calculated from the full model, regardless of the significances of main effects or interactions.

Assumptions of normality and homogeneity of variances were determined using Shapiro–Wilk test, diagnostic plots and Levene’s test with plotresid() function in the *RVAideMemoire* package [45] and leveneTest() function in the *car* package [46]. If assumptions were not met, boxcox and rank transformation were performed to achieve normality (using boxcoxnc() function in the *AID* package [47], and rank() function). In all cases, the results remained unchanged. Hence, we retained non-transformed data in all final analyses for ease of interpretation.

## 3. Results

All raw values for mitochondrial respiration rates and FCEs between assay temperatures among all treatments are represented in electronic supplementary material, figures S3 and S4.

### (a) Effect of physiological hyperthermia on mitochondrial function

**Figure 2.**
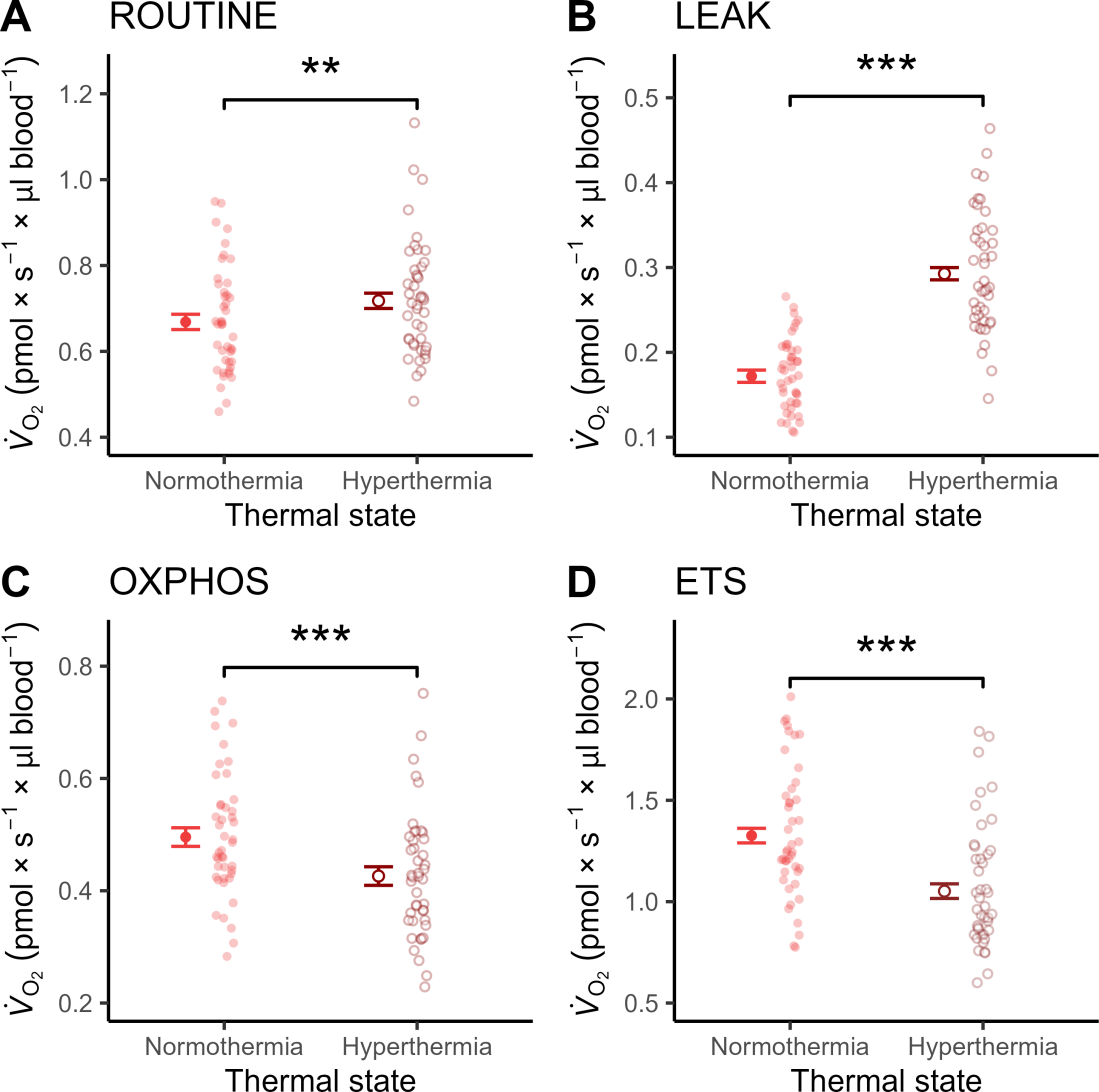
Mitochondrial respiration rates at representative normothermic (41°C) and hyperthermic (45°C) body temperatures in whole blood of 12-week-old Japanese quail. Points and error bar show estimated means ± standard error (s.e.) from the final models, and semi-transparent points show raw data. Asterisks indicate level of significance when comparing experimental treatments: **p < 0.01; ***p < 0.001). ${\dot{V}}_{O_{2}}$: oxygen consumption.

**Figure 3.**
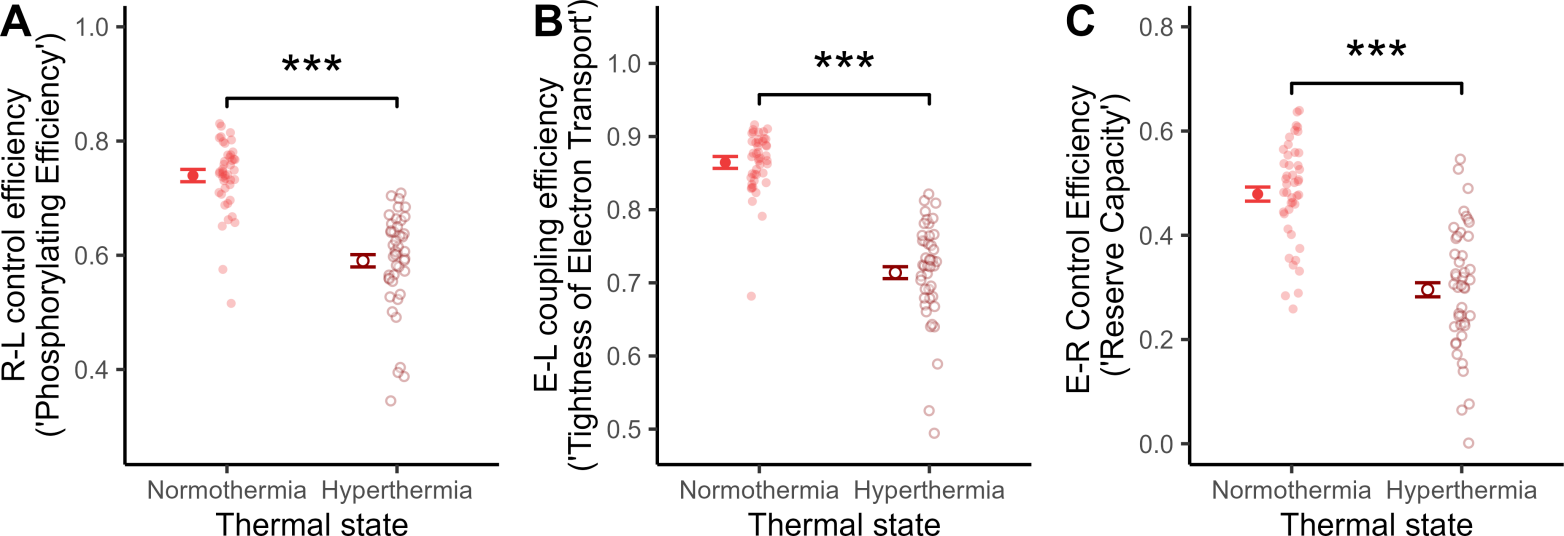
Mitochondrial flux control efficiencies (FCEs) at representative normothermic (41°C) and hyperthermic (45°C) body temperatures in whole blood of 12-week-old Japanese quail. Points and error bar show estimated means ± s.e. from the final models, and semi-transparent points show raw data. Asterisks indicate level of significance between experimental treatments: ***p < 0.001.

ROUTINE (i.e. the baseline respiration on endogenous substrates) increased significantly at the heat-stressed assay temperature (i.e. 45°C), by 1.07-fold compared to at a representative body temperature (41°C) (LMM: *p =* 0.004; figure 2A). This was mainly due to 1.70-fold increase in LEAK (i.e. respiration used to counteract proton leak) (*p <* 0.001), whereas OXPHOS (i.e. ATP-producing respiration) was 0.86-fold lower at 45°C (*p =* 0.001) (figure 2B,C). ETS (i.e. the maximum capacity of the electron transport system) was also significantly lower at a heat stressed assay temperature, by 0.79-fold (*p <* 0.001) (figure 2D). As a result, all FCEs (see electronic supplementary material, table S2 for definitions) indicated an overall decrease in mitochondrial efficiency during simulated heat stress (figure 3A–C; all *p <* 0.001; table 1). Whatever the assay temperature, females displayed significantly higher values of all mitochondrial respiration traits and FCEs compared with males, except for OXPHOS and R-L control efficiency where males and females did not differ (table 1; electronic supplementary material, figures S5 and S6).

**Table 1.** Final model estimates (±standard error), degrees of freedom, test statistics and *p*-values when testing the effects of assay temperature (41°C and 45°C) amongst treatments (Cold, Warm, Cold-mild and Warm-mild) on mitochondrial respiration in intact blood cells of 12-week-old Japanese quail. Significant effects (*p* ≤ 0.05) are printed in bold font and near-significant effects (0.05 > *p* ≤ 0.10) are printed in italics. birdID: unique identifier of each bird; s.e.: standard error; d.f.: degrees of freedom; σ_ID_: standard deviation of the random effect; σ_resid_: residual standard deviation.

models and parameters	estimate (± s.e.)	d.f.	*F*	*p*	σ_ID_	σ_resid._
ROUTINE (pmol O_2 _× s^−1^ × µl blood^−1^)						
assay temperature × treatment		3, 40	0.582	0.630		
assay temperature		1, 40	9.140	**0.004**		
41°C	0.669 (±0.018)					
45°C	0.718 (±0.018)					
treatment		3, 40.7	0.657	0.583		
sex		1, 39	10.351	**0.003**		
female	0.744 (±0.021)					
male	0.642 (±0.024)					
birdID		1		**<0.0001**	0.089	0.076
OXPHOS (pmol O_2 _× s^−1^ × µl blood^−1^)						
assay temperature × treatment		3, 40	1.842	0.155		
assay temperature		1, 40	14.607	**0.001**		
41°C	0.497 (±0.016)					
45°C	0.425 (±0.016)					
treatment		3, 40.4	1.860	0.152		
sex		1, 39	3.578	*0.066*		
birdID		1		**0.026**	0.064	0.088
LEAK (pmol O_2 _× s^-1^ × µl blood^-1^)						
assay temperature × treatment		3, 40	2.881	**0.048**		
cold	0.040 (±0.004)					
warm	0.025 (±0.004)					
cold-mild	0.029 (±0.004)					
warm-mild	0.026 (±0.004)					
assay temperature		1, 40	223.05	**<0.0001**		
41°C	0.172 (±0.007)					
45°C	0.293 (±0.007)					
treatment		3, 40.4	2.455	*0.077*		
sex		1, 39	18.175	**0.0001**		
female	0.258 (±0.008)					
male	0.207 (±0.008)					
birdID		1		**0.016**	0.029	0.038
ETS (pmol O_2 _× s^-1^ × µl blood^-1^)						
assay temperature × treatment		3, 40	0.754	0.527		
assay temperature		1, 40	85.982	**<0.0001**		
41°C	1.33 (±0.04)					
45°C	1.05 (±0.04)					
treatment		3, 40.9	0.913	0.443		
sex		1, 39	39.924	**<0.0001**		
female	1.40 (±0.04)					
male	0.98 (±0.05)					
birdID		1		**<0.0001**	0.194	0.138
R–L control efficiency						
assay temperature × treatment		3,40	2.211	0.102		
assay temperature		1,40	104.361	**<0.0001**		
41°C	0.74 (±0.011)					
45°C	0.59 (±0.011)					
treatment		3,40.2	2.054	0.122		
sex		1, 39		0.121		
birdID		1		0.628	0.019	0.068
E–L coupling efficiency						
assay temperature × treatment		3, 40	1.731	0.176		
assay temperature		1, 40	211.917	**<0.0001**		
41°C	0.865 (±0.008)					
45°C	0.714 (±0.008)					
treatment		3, 40.3	1.639	0.195		
sex		1, 39	4.253			
female	0.802 (±0.008)					
male	0.776 (±0.009)					
birdID		1		0.237	0.023	0.048
E–R control efficiency						
assay temperature × treatment		3, 40	0.099	0.960		
assay temperature		1, 40	165.786	**<0.0001**		
41°C	0.479 (±0.014)					
45°C	0.295 (±0.014)					
treatment		3, 40.4	0.082	0.969		
sex		1, 39	29.959	**<0.0001**		
female	0.450 (± 0.016)					
male	0.324 (± 0.017)					
birdID		1		**0.003**	0.059	0.067

### (b) Effects of postnatal temperature on the thermal sensitivity of mitochondrial function

**Figure 4.**
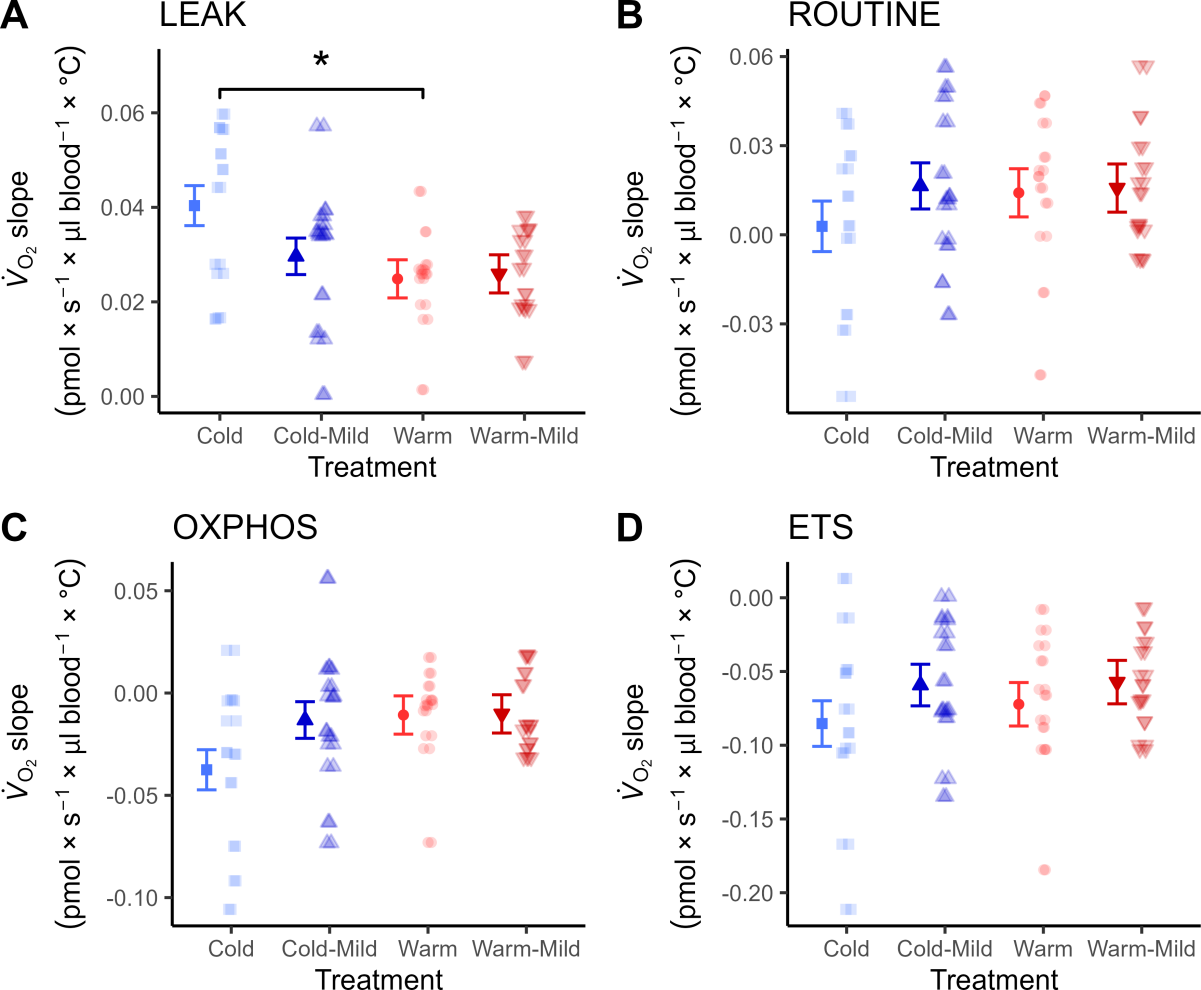
Change in mitochondrial respiration per degree C increase between a normothermic (41°C) and a hyperthermic (45°C) assay temperature (${\dot{V}}_{O_{2}}$slope) in whole blood of Japanese quail. The birds were raised in Cold (10°C) or Warm (30°C) environments from hatching until 12 weeks post hatch (wph) or were transferred from these conditions into a common garden (20°C; Cold-mild, Warm-mild) at 9 wph. Data were collected from all groups at 12 wph. Points and error bars how estimated mean trends ± s.e., and semi-transparent points show individual trends. Asterisks indicate level of significance between experimental treatments (*p < 0.05) within relevant post hoc comparisons: Cold versus Warm, Cold versus Cold-mild, Warm versus Warm-mild and Cold-mild versus Warm-mild.

The thermal sensitivity of LEAK (i.e. the increase in respiration between *ex vivo* normothermia and hyperthermia) was 1.62-fold higher in the Cold compared with the Warm birds (figure 4A) but did not differ between any of the other treatment contrasts. There were no differences in thermal sensitivity between developmental temperature treatment for any of the other respiration traits considered (figure 4B–D, table 1).

### (c) Effects of postnatal developmental temperature and body mass and size

Body mass was not affected by postnatal temperature (electronic supplementary material, figures S7A, table S3). However, Warm birds had significantly longer wings (124.26 ± 1.02 mm) compared with Cold birds (120.20 ± 1.06 mm; LM: *p =* 0.01) at 12 wph (electronic supplementary material, figures S7B, table S3). Moreover, females were heavier and longer winged across treatments compared with males (electronic supplementary material, figure S7C–D, table S3).

## 4. Discussion

Physiological hyperthermia simulating a body temperature that quail regularly incur during heat stress, caused significantly higher mitochondrial respiration (ROUTINE) in intact blood cells. This was driven by an increase in LEAK, which was 1.7-fold higher at 45°C compared with 41°C (figure 2A,B). ATP-producing respiration (OXPHOS), on the other hand, dropped significantly during hyperthermia (figure 2C). Additionally, maximum working capacity of the mitochondria (ETS) was significantly lower during heat stress (figure 2D). In aggregate, when the blood was exposed to hyperthermia, the phosphorylating capacity in the baseline state (R–L control efficiency), coupling efficiency in a stimulated state (E–L coupling efficiency), and mitochondrial aerobic scope (E–R control efficiency), were all significantly lower than in normothermia (figure 3A–C).

Acute heat stress increases the production of reactive oxygen species (ROS) and decreases antioxidant defences (reviewed by [48,49]). Increased proton leak in a hyperthermic cell could therefore be beneficial since it reduces ROS production [50,51]. This, in turn, may improve cell survival during a heat challenge [52]. Any such benefits must, however, be leveraged against the potentially harmful thermal effects of increased LEAK, which will add additional heat to an already heat-stressed body [53]; a consequence that can hardly be viewed as adaptive. Our protocol cannot reveal if the effect on LEAK was due to passive thermal effects acting on membrane fluidity or active upregulation of proton conductance (cf. [54]). While research on mammals show that there is a regulated increase proton leak in heat-challenged cells [55], avian studies suggest that the expression of putative uncoupling proteins (avUCP, ANT) is lower or unaltered during heat stress [56,57]. The precise mechanisms underpinning increased LEAK in hyperthermic cells, whether regulated by uncoupled proteins (cf. [54]) or emergent from direct thermal effects acting on membrane fluidity (cf. [25]), should be investigated in further studies.

The reductions in both OXPHOS and ETS observed in the hyperthermic condition indicate that heat stress may come at a cost of reduced ATP production, as previously demonstrated in ectotherms [27]. This is in contrast to a recent study of physiological hypothermia, where a corresponding reduction in assay temperature revealed no effects of temperature on neither OXPHOS nor ETS [25]. Thus, the thermal sensitivity of mitochondrial respiration traits appears to be nonlinear over a physiologically relevant range of body temperatures that most birds experience over the course of their annual cycle. However, the effect of hyperthermia on OXPHOS might be tissue dependent. For example, previous research on both birds and mammals found no significant change in phosphorylating respiration in permeabilized skeletal muscle cells during *ex vivo* or *in vitro* hyperthermia [28,55]. Alternatively, lower OXPHOS and ETS in hyperthermic blood cells could present if intracellular substrate quantity is insufficient to meet the higher metabolic requirements caused by rising temperature *per se*. It would thus be interesting to repeat the experiment using permeabilized blood cells to separate the potentially limiting effects of substrate availability [34] from direct thermal effects acting on the mitochondrial complexes (e.g. [58]).

Cold-reared birds displayed a greater increase in LEAK compared with warm-acclimated birds upon physiological hyperthermia, suggesting that postnatal developmental temperature influences the thermal sensitivity of blood cell mitochondria (figure 3A). These results could be explained if cold-induced changes in mitochondrial structure [59] or membrane fluidity [60], rendered mitochondrial metabolism more sensitive to an acute heat challenge. Mechanisms aside, our work indicates that acclimation to cold temperature at the organismal level might not be compatible with maintained heat tolerance at the level of cell function. This could be relevant for understanding the apparent evolutionary trade-off between reproductive heat- and cold-tolerance that was recently demonstrated in birds [61].

Thermal sensitivity did not vary between the Cold-mild and Warm-mild birds after three weeks in a common garden. Moreover, thermal sensitivity had shifted downwards in absolute terms when comparing the Cold-mild and Cold birds, and it did not differ between the Warm and Warm-mild birds. From this, we can infer both that exposure to cold temperatures constrains mitochondrial heat tolerance but also that the cold-induced phenotype did not reflect irreversible developmental programming. We have recently found similar reversible plasticity at the level of organismal thermoregulation in response to postnatal developmental temperature manipulation in quail [9]. While reversible developmental plasticity may be more common than often appreciated [62], these results contrast both to studies that manipulated temperature during the embryonic period in precocial birds [23] and those subjecting altricial nestlings to an intense heatwave during the early post-hatching life [20,24], although it should be noted that neither of these experiments addressed the mitochondrial *response* to a thermal stressor. It is possible that quail, which hatch with comparatively advanced thermogenic and thermolytic capacity, are more amenable for ontogenetic programming of energy metabolism by conditions experienced *before* hatching.

Several studies suggest that birds handle heat challenges better when exposed to warmth during early life stages (e.g. [10,11,63,64]). However, at the cellular level, growing up in a warm environment did not lead to significant improvements in the heat tolerance of mitochondrial function compared with the common garden birds that had spent three weeks at a mild temperature before the measurements. This could be related to the choice of acclimation temperature, as 30°C is within the thermoneutral zone of quail [9,65]. Yet, quail reared in 30°C displayed (reversible) improved evaporative cooling capacity, a proxy for heat tolerance, compared with quail reared at mild temperature (20°C), whereas cold-acclimation (10°C) did not lead to any reductions in evaporative capacity in response to a mild heat challenge [9]. A comparable mismatch between organismal and cellular responses has previously been observed in response to other environmental stressors across taxa, such as oxygen availability in goldfish *Carassius auratus* [66]. Our results therefore suggest that even when thermoregulatory responses are indistinguishable at the organismal level, cold-adapted birds might accrue somatic costs at a higher rate upon environmental heating, since their cells cannot keep pace. While this notion needs confirmation *in vivo* and over a range of metabolically relevant tissues, our *ex vivo* findings emphasize the need to investigate effects of hot weather across levels of organization. As a final remark, it is noteworthy that warm acclimation had small, if any, effects on thermal sensitivity of mitochondria compared with acclimation to a mild, average temperature. This raises concerns that, much like in ectotherms [67,68], warm-blooded endotherms may have a small scope to improve the physiological tolerance of somatic function in a warming world, increasing their reliance on behavioural thermoregulation with downstream missed-opportunity costs and loss of fitness.

## Data Availability

Supporting data and code are deposited in Figshare [69]. Supplementary material is available online [70].
